# Multiple Adenomatous Duodenal Polyposis

**DOI:** 10.1155/2013/181704

**Published:** 2013-03-31

**Authors:** Zdena Zádorová, Jan Hajer, Václav Mandys

**Affiliations:** ^1^2nd Department of Internal Medicine,Third Faculty of Medicine, Charles University in Prague, Šrobárova 50 100 34, Prague 10, Czech Republic; ^2^Department of Pathological Anatomy, Third Faculty of Medicine, Charles University in Prague, Šrobárova 50 100 34, Czech Republic

## Abstract

Multiple duodenal polyps are a relatively rare finding, usually co-occurrent with familial adenomatous polyposis (FAP).We report a patient with multiple duodenal adenomas and a negative examination for FAP: multiple flat polyps were detected endoscopically in a 37-year-old male patient, extending from the apex of the bulb to the end of the descending part of the duodenum. In terms of histology, they were tubular adenomas with moderate dysplasia. Colonoscopy and enteroclysis were normal. Both push and capsule enteroscopy only showed multiple polyps in the area of the descending duodenum. DNA analysis of the APC gene was as follows: DGGE, exon 1–15, deletion at codons 1309 and 1061 by means of PCR for attenuated APC were negative. Afterwards we screened the patient for germline MYH mutations using the denaturing high-performance liquid chromatography (DHPLC) in combination with sequencing. No novel pathogenic mutation has been identified. Large polyps were removed by means of endoscopic polypectomy and mucosectomy, while small polyps were removed by means of argon plasma coagulation.We conduct yearly checkups, removing only sporadic polyps. The rare finding of duodenal polyposis not co-occurrent with FAP proves that multiple adenomas in the digestive tube need not necessarily co-occur with FAP.

## 1. Introduction

Duodenal polyps are usually found in up to 4.6 percent of patients undergoing endoscopy of the upper part of the digestive tube [1]. Mostly adenomas, these polyps are the least frequent ones encountered in the descending duodenum (accounting for <0.05 percent of all intestinal adenomas) [2]. Most adenomas occur near the ampulla of Vater. Patients usually range between 30 and 90 years of age, with the highest incidence in the seventh decade [2]. Most small adenomas are asymptomatic and are usually detected accidentally by endoscopy of the upper part of the gastrointestinal tract conducted for another reason. Multiple duodenal adenomas are a rare finding, usually cooccurring with familial adenomatous polyposis (FAP) or attenuated familial adenomatous polyposis (AFAP). This special form of familial polyposis, also referred to in the literature as the hereditary flat adenoma syndrome, is due to a mutation in the proximal part of the APC gene [3, 4]. The actual incidence and frequency of AFAP are unknown [5]. The chief manifestation of this phenotypically different form of familial adenomatous polyposis is in flat adenomas of the large bowel that appear in small numbers (up to 100 polyps) [3]. In most cases, polyps of the gastric body, due to dilatation of fundic glands, are present, while the literature also describes adenomas of the duodenum [4, 5]. Some of the APC negative FAP and AFAP cases have recently been found to be attributable to MYH-associated polyposis (MAP). MAP is autosomal recessive syndrome associated with 5–100 colorectal adenomas and caused by mutation in the MYH gene [6].

## 2. Case Report

**Figure 1:**
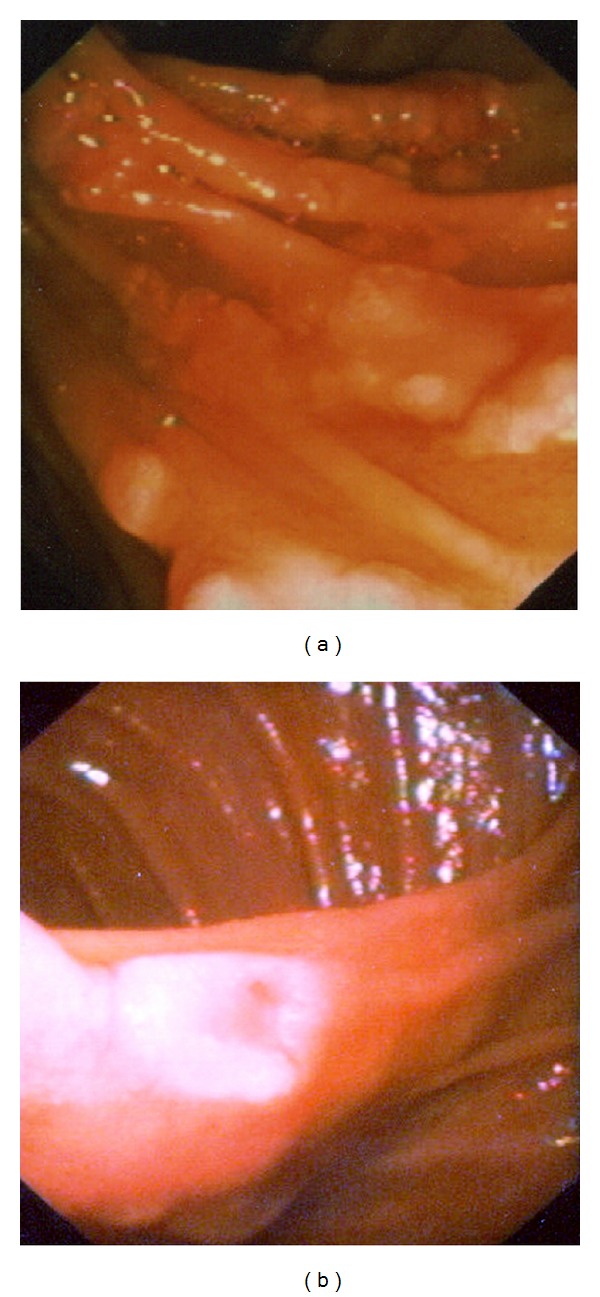
(a) Multiple (several dozen) irregular flat polyps in area of the descending duodenum, in locations other than the ampulla of Vater. (b) Multiple irregular flat polyps from 5 mm to approximately 15 mm in size, rather atypical in appearance with a central depression.

**Figure 2:**
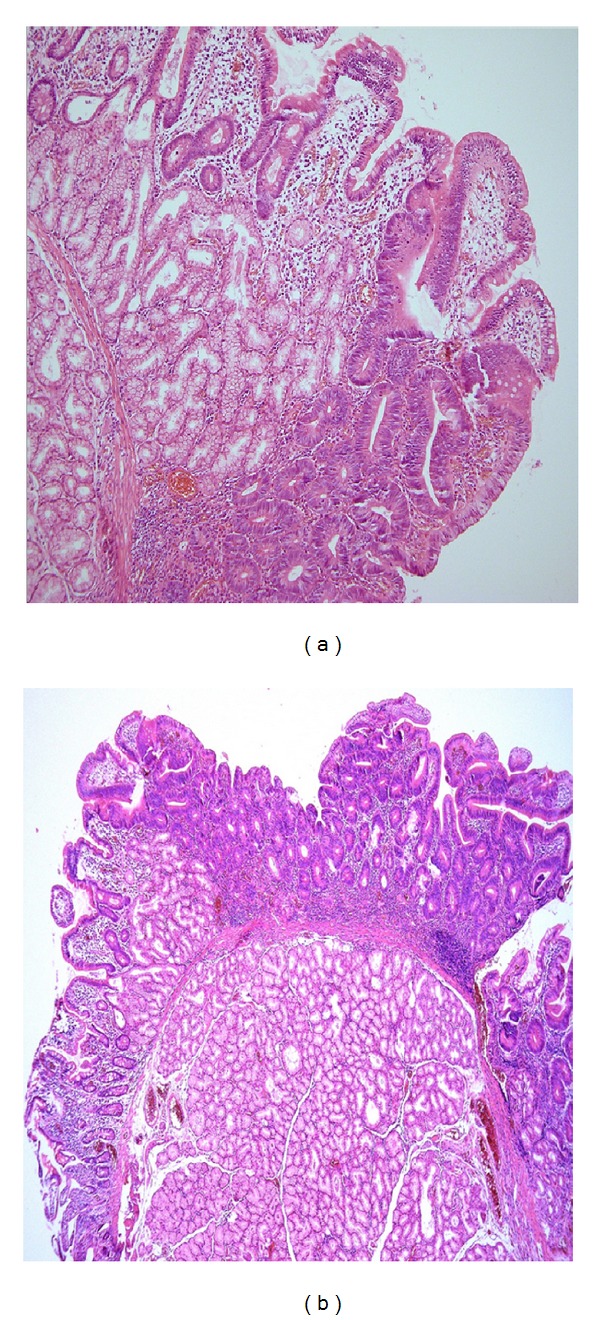
(a) Histological examination of lesions showed structures of the tubular or tubulovillous adenoma with mildly to moderately dysplastic epithelium and numerous Paneth cells. (b) Flat character of adenoma with central depression.

In 2008, a 37-year-old male patient was examined at our institution for irregular postprandial dyspeptic complaints and pain in the epigastrium which had lasted for several months and receded spontaneously. Alternating stools occurred intermittently. Family medical history was insignificant and the patient had never suffered a serious illness. Neither physical nor laboratory examination revealed any pathological findings. Gastroscopic examination revealed multiple (several dozen) irregular flat polyps from 5 mm to approximately 15 mm in size, rather atypical in appearance with a central depression in the area of the descending duodenum, in locations except the ampulla of Vater (Figures 1(a) and 1(b)). Histological examination of these lesions showed structures of the tubular or tubulovillous adenoma with mildly to moderately dysplastic epithelium and numerous Paneth cells (Figures 2(a) and 2(b)).

Since we assumed that the patient might suffer from FAP, he was first subjected to colonoscopy, the results of which were normal. This examination was followed by push enteroscopy, which confirmed the presence of polyps in the area of the duodenum only. Capsule enteroscopy ruled out the presence of polyps in the aboral part of the small bowel. 

Subsequently, a genetic examination for AFAP was initiated. A DNA analysis with direct detection of the most common mutations in the APC gene, using the PCR technique, did not confirm 5 bp deletion at codons 1309 and 1061 of the APC gene. Direct diagnosis of mutations in the APC gene using the DGGE method did not confirm a mutation in the screened part—exons 1–14 and exon 15 (approximately up to codon 1770)—of the APC gene. Afterwards we screened the patient for germline MYH mutations using the denaturing high-performance liquid chromatography (DHPLC) in combination with sequencing. No novel pathogenic mutation has been identified. Over several sessions, the polyps were removed endoscopically—large ones by means of mucosectomy and small ones by means of argon plasma coagulation. Following the endoscopic therapy, we checked the patient regularly each six months in the first year; at present, he comes for a checkup once a year. In each successive session, several (up to five) very small adenomas were found, which were removed using argon plasma coagulation. During the last checkup this year, two small polyps up to 4 mm in size were removed. The patient has no subjective complaints; the successive laboratory results are within the norm.

## 3. Discussion

Multiple duodenal adenomas are usually found in patients with FAP, AFAP, or MAP, while the literature suggests that nonfamilial adenomatous polyposis in this area is a rare finding [7]. Instead, the literature more frequently describes solitary polyps in the duodenum and the techniques for their management, either endoscopic or surgical [8]. These polyps are usually asymptomatic; only adenomas in the ampullar location may cause symptoms of a biliary disorder [2]. Large duodenal adenomas may manifest themselves through dyspeptic complaints or bleeding into the upper part of the digestive tube. In this area, sessile adenomas are described more often than pedunculated ones [2].

It is unknown whether patients with duodenal adenomas are at greater risk for neoplasia in the small bowel. With the increasing availability of capsule and endoscopic enteroscopy, papers have appeared that investigate the incidence of polyps in the small bowel [9]. Rieman's results show that the incidence of small polyps in patients with duodenal adenomas without FAP is higher than in a control group which underwent capsule endoscopy for another reason. Most patients with the finding of duodenal adenomas undergo a colonoscopic examination, and the risk of a simultaneous occurrence of adenomas in the large bowel seems to be higher [10–13].

Treatment of duodenal adenomas depends on their location, size, and degree of dysplasia. In addition to standard surgical resection, the literature describes other options of endoscopic therapy—polypectomy or mucosectomy, as well as argon plasma coagulation—that show good results and few complications. Due to a relatively frequent recurrence of adenomas, patients require long-term observation [8, 14]. In our patient, it was possible to remove the large adenomas using a polypectomy snare and the small ones using argon plasma coagulation. The number of polyps has not increased between the yearly checkups; we have only removed sporadic polyps up to 4 mm in size.
